# Transcriptome Sequencing Reveals Potential Mechanism of Cryptic 3’ Splice Site Selection in *SF3B1*-mutated Cancers

**DOI:** 10.1371/journal.pcbi.1004105

**Published:** 2015-03-13

**Authors:** Christopher DeBoever, Emanuela M. Ghia, Peter J. Shepard, Laura Rassenti, Christian L. Barrett, Kristen Jepsen, Catriona H. M. Jamieson, Dennis Carson, Thomas J. Kipps, Kelly A. Frazer

**Affiliations:** 1 Bioinformatics and Systems Biology, University of California San Diego, La Jolla, California, United States of America; 2 Moores Cancer Center, University of California San Diego, La Jolla, California, United States of America; 3 Department of Pediatrics and Rady Children's Hospital, University of California San Diego, La Jolla, California, United States of America; 4 Institute for Genomic Medicine, University of California San Diego, La Jolla, California, United States of America; 5 Department of Medicine, University of California San Diego, La Jolla, California, United States of America; 6 Sanford Consortium for Regenerative Medicine, University of California San Diego, La Jolla, California, United States of America; National Research Council of Canada, Canada

## Abstract

Mutations in the splicing factor *SF3B1* are found in several cancer types and have been associated with various splicing defects. Using transcriptome sequencing data from chronic lymphocytic leukemia, breast cancer and uveal melanoma tumor samples, we show that hundreds of cryptic 3’ splice sites (3’SSs) are used in cancers with *SF3B1* mutations. We define the necessary sequence context for the observed cryptic 3’ SSs and propose that cryptic 3’SS selection is a result of *SF3B1* mutations causing a shift in the sterically protected region downstream of the branch point. While most cryptic 3’SSs are present at low frequency (<10%) relative to nearby canonical 3’SSs, we identified ten genes that preferred out-of-frame cryptic 3’SSs. We show that cancers with mutations in the *SF3B1* HEAT 5-9 repeats use cryptic 3’SSs downstream of the branch point and provide both a mechanistic model consistent with published experimental data and affected targets that will guide further research into the oncogenic effects of *SF3B1* mutation.

## Author Summary

A key goal of cancer genomics studies is to identify genes that are recurrently mutated at a rate above background and likely contribute to cancer development. Many such recurrently mutated genes have been identified over the last few years, but we often do not know the underlying mechanisms by which they contribute to cancer growth. Unexpectedly, several genes in the spliceosome, the collection of RNAs and proteins that remove introns from transcribed RNAs, are recurrently mutated in different cancers. Here, we have examined mutations in the splicing factor *SF3B1*, a key component of the spliceosome, and identified a global splicing defect present in different cancers with *SF3B1* mutations by comparing the expression of splice junctions using generalized linear models. While prior studies have reported a limited number of aberrant splicing events in *SF3B1-*mutated cancers, we have established that *SF3B1* mutations are associated with usage of hundreds of atypical splice sites at the 3’ end of the intron. We have identified nucleotide sequence requirements for these cryptic splice sites that are consistent with a proposed mechanistic model. These findings greatly expand our understanding of the effect of *SF3B1* mutations on splicing and provide new targets for determining the oncogenic effect of *SF3B1* mutations.

## Introduction

One of the biggest surprises to emerge from the growing catalog of somatic mutations in various cancer types is the recurrent mutation of genes encoding the RNA spliceosome [1]. Recurrent mutations in the highly conserved HEAT 5–9 repeats of splicing factor 3B subunit 1 (*SF3B1*) have been reported in myelodysplastic syndrome, chronic lymphocytic leukemia (CLL), breast cancer (BRCA), uveal melanoma (UM), and pancreatic cancer [2–7]. *SF3B1* mutation is associated with poor prognosis in CLL but improved prognosis in myelodysplasia and UM [2,7–9]. Prior studies have shown that mutated *SF3B1* CLL samples have differential exon inclusion and use some cryptic 3’ splice sites (3’SSs) relative to wild-type *SF3B1* CLL samples [5,6,8,10,11]. However, it is unknown whether *SF3B1* mutation is associated with the same 3’SS selection defects in different cancers. The mechanism underlying the cryptic 3’SS selection and the functional consequences thereof remain unresolved as well.

SF3B1 is a core part of the U2-small nuclear ribonucleoprotein (U2-snRNP) complex and stabilizes the binding of the U2-snRNP to the branch point (BP), a degenerate sequence motif usually located 21–34 bp upstream of the 3’SS [12,13]. SF3B1 also interacts with other spliceosomal proteins such as U2AF2, which binds the polypyrimidine tract (PPT) downstream of the BP [2,14,15]. The binding of the U2-snRNP and other spliceosome proteins around the BP prevents 3’SS selection in a ~12–18 bp region directly downstream of the BP due to steric hindrance [16,17]. Inherited *cis*-acting splicing mutations beyond this ~12–18 bp region downstream of the BP that result in the use of cryptic 3’SSs have been shown to occur in Mendelian disease genes [18]. Additionally, a competitive region exists ~12 bp downstream from the first 3’SS after the protected region where AG dinucleotides can compete to be used as 3’SSs based on sequence characteristics such as the PPT length, distance from the BP, nucleotide preceding the AG dinucleotide, and other features [17].

The role of SF3B1 and the U2-snRNP in recognizing and binding the BP and the localization of mutations to HEAT 5–9 repeats suggest that *SF3B1* mutations are dominant drivers that may alter 3’SS selection [6]. To test this, we examined splice site usage in transcriptome data from *SF3B1* mutant and *SF3B1* wild-type CLL, UM and BRCA cases. We identified 619 cryptic 3’SSs used more frequently in *SF3B1* mutants and clustered 10–30 bp upstream of canonical 3’SSs. The majority of these cryptic 3’SSs were observed in all three tumor types despite the divergent clinical implications of *SF3B1* mutation. Our analysis of tumors with *SF3B1* mutations shows that cryptic 3’SS selection occurs only in samples with missense mutations at ~10 amino acid hotspots in the fifth to ninth HEAT repeats. We analyzed the organization of splicing motifs around the cryptic 3’SSs and found that only introns with an AG dinucleotide at the boundary of the sterically protected region downstream of the BP but >10 bp upstream of the canonical 3’SS are susceptible to cryptic 3’SS selection in *SF3B1* mutants. We assessed the functional impact of *SF3B1* mutation and found that the cryptic 3’SSs are typically used at low frequency in the *SF3B1* mutants (<10% relative to the canonical splice site) and are sometimes present in the *SF3B1* wild-types but at an even lower frequency (<0.5% relative to the canonical splice site). However, we identified 10 candidate genes, some previously implicated in tumorigenesis, for which there is a high amount of out-of-frame cryptic splice site usage that may affect the function of these genes.

## Results

### Cryptic 3’ splice sites 10–30 bp upstream of canonical 3’ splice sites are used in *SF3B1* mutants

**Fig 1:**
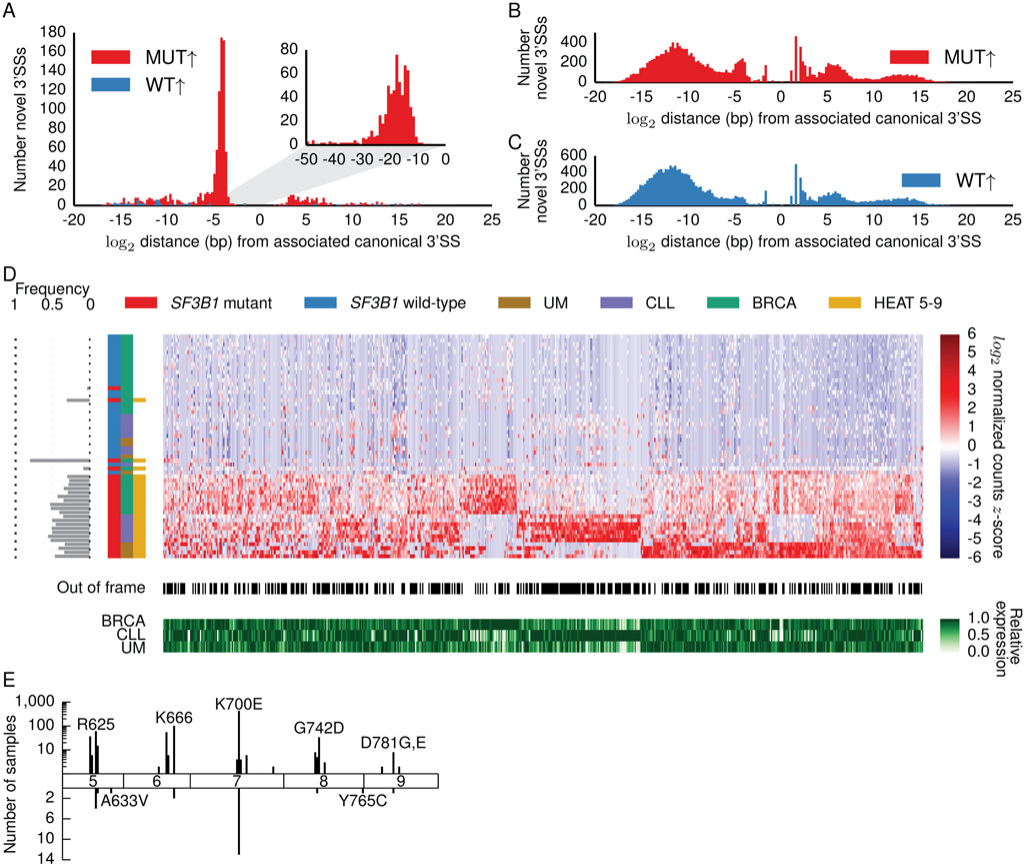
Proximal cryptic 3’SSs used significantly more often in cancers with *SF3B1* hotspot mutations. log_2_ distance in base pairs from associated canonical 3’SSs to (A) 1,117 significantly differentially used novel 3’SSs, (B) 16,673 novel 3’SSs with canonical intron motifs (GT/AG) used more highly in the mutants but not significant, and (C) 18,660 novel 3’SSs with canonical intron motifs (GT/AG) used more highly in the wild-types but not significant. Zero represents the position of the canonical 3’SS. Negative and positive distances indicate that the cryptic 3’SS is respectively upstream or downstream from the canonical 3’SS. Inset in (A) shows base-by-base binning from zero to 50 base pairs upstream of canonical 3’SS. Red and blue histograms represent junctions with significantly higher usage in *SF3B1* mutants or *SF3B1* wild-type samples, respectively. (D) Upper red and blue heatmap shows for each sample the log_2_ library-normalized count *z*-score for 619 cryptic 3’SSs used significantly more often in the *SF3B1* mutants and located 10–30 bp upstream of canonical 3’SSs (DEXSeq, BH-adjusted *p* < 0.1). Grey bars at left indicate frequency of *SF3B1* mutant allele in RNA-seq data. Colorbars indicate *SF3B1* mutation status, cancer type, and whether the *SF3B1* mutation is located in the HEAT 5–9 repeats. Black and white colorbar indicates whether novel 3’SSs are out-of-frame (black) relative to canonical 3’SSs. Bottom green heatmap shows relative expression levels for the genes containing each cryptic 3’SS. We calculated the average expression of each gene in each cancer type and normalized by the maximum expression for each gene so that the maximum value in each column is one (see Methods). Cryptic 3’SSs not observed in all cancer types tend to have differing gene expression levels between cancers. (E) Locations and frequency of *SF3B1* mutations in HEAT repeats 5–9. Mutations observed more than once in COSMIC (upper axis) cluster in ~10 amino acid hotspots in each HEAT repeat; most frequent mutation in each hotspot is labeled. Bottom axis shows locations and frequency of mutations in our study. BRCA samples with A663V and Y765C mutations do not show evidence for cryptic 3’SS selection.

We used RNA-sequencing data from *SF3B1* mutated and *SF3B1* wild-type chronic lymphocytic leukemia (CLL; seven mutant, nine wild-type), breast cancer (BRCA; 14 mutant, 18 wild-type), and uveal melanoma (UM; four mutant, four wild-type) samples (S1 Fig., S1 File) to test 219,476 splice junctions present in the Gencode v14 gene annotation [19] along with 87,941 novel splice junctions (not annotated in Gencode) for differential usage by comparing junction-spanning reads using a generalized linear model as implemented in DEXSeq [20]. A splice junction is considered differentially used between mutant and wild-type samples if the expression level of that junction differs significantly after accounting for overall expression differences of the corresponding gene locus. All tested junctions were covered by at least 20 reads summed over all cancer samples in a given analysis, shared a 5’ splice site and/or 3’SS with a Gencode splice junction, and had a known splice site motif. We identified 1,749 junctions that were significantly differentially used between the *SF3B1* mutant and *SF3B1* wild-type samples across the three tumor types including 1,330 novel junctions, of which 1,117 are novel 3’SSs (BH-adjusted *p* < 0.1, S2 File). These 1,749 significant junctions were highly enriched for novel splice junctions compared to annotated junctions (Fisher exact, *p <* 10^-200^) and the novel junctions were enriched for novel 3’SSs (Fisher exact, *p <* 10^-200^) showing that *SF3B1* mutations result in the usage of a large number of novel 3’SSs. These 1,749 significant junctions include 61 of 79 splice sites recently reported as specific to CLL cases with *SF3B1* mutations [11] supporting the specificity of our approach while demonstrating an increased sensitivity that has allowed us to identify many more cryptic 3’SSs than previously reported. We plotted the distance between each significant novel 3’SS and its associated canonical 3’SS (defined as the nearest Gencode 3’SS that shared the same 5’ splice site—see Methods). Of the 1,117 significant novel 3’SSs, 619 were proximal cryptic 3’SSs clustered 10–30 bp upstream of their associated canonical 3’SSs while the remaining 498 cryptic 3’SSs were widely distributed (herein referred to as distal cryptic 3’SSs) (Fig. 1A, S3 File). All of the 619 proximal cryptic 3’SSs were used more often in the *SF3B1* mutant samples compared to the wild-type samples and 58% were out-of-frame relative to the nearby canonical 3’SSs, suggesting that these are not canonical 3’SSs missing from Gencode. 417 of the 498 distal cryptic 3’SSs were also used more highly in the *SF3B1* mutants (S4 File). The distribution of the 1,117 significant novel 3’SSs is different from that of novel 3’SSs whose usage did not differ significantly between the *SF3B1* mutants and wild-types (Fig. 1B,C), further demonstrating that the usage of proximal cryptic 3’SSs is a property of *SF3B1* mutants. Examining each tumor type individually, we observed the same enrichment of cryptic 3’SSs 10–30 bp upstream of canonical splice sites (S2 Fig.). Given these observations, SF3B1’s role in binding the BP, and the organization of the BP and splicing motifs in the last 30 bp of the intron [12], we focused our initial analyses on the 619 proximal cryptic 3’SSs.

### Cryptic 3’SS selection is limited to tumors with mutations in HEAT repeat hotspots

We clustered all samples based on the read coverage of the 619 proximal cryptic 3’SSs and found that four *SF3B1*-mutated BRCA samples did not cluster with the other mutants (Fig. 1D). The *SF3B1* mutation for one of these BRCA samples was a nonsense mutation not located in the HEAT 5–9 repeats while another sample had a subclonal (8.4%) HEAT 5–9 mutation with attenuated cryptic 3’SS selection (S3 Fig.). The other two samples had mutations in the HEAT 5–9 repeats but outside of the apparent ~10 amino acid mutational hotspots (Fig. 1E). We observed cryptic 3’SS selection in a TCGA lung adenocarcinoma sample with a hotspot mutation but not in lung cancer samples with *SF3B1* mutations outside of the five hotspots (S4 Fig.). These results show that cryptic 3’SS selection only occurs in tumors carrying mutations in one of the five ~10 amino acid hotspots in the HEAT 5–9 repeats and is not limited to cancers in which *SF3B1* is recurrently mutated.

### Cryptic 3’SSs are shared across different cancer types

The majority of the 619 proximal cryptic 3’SSs were used in *SF3B1*-mutated samples in all three cancer types suggesting that the mechanism of cryptic 3’SS selection in *SF3B1*-mutated tumors is the same between different cancers (Fig. 1D). Some cryptic 3’SSs were not used in one or two of the cancer types due to lower expression of the corresponding genes in those cancers. Differences in cryptic 3’SS usage due to varying gene expression may contribute to the divergent prognostic implications of *SF3B1* mutation in various cancers [2,7].

To characterize the roles of the genes affected by cryptic 3’SS usage, we performed a gene set enrichment analysis for the 912 genes that contained the 619 proximal and 417 distal cryptic 3’SSs used significantly more often in the *SF3B1* mutant samples (S5 File). The gene set with the second smallest *p*-value consists of genes up-regulated in chronic myelogenous leukemia and the seventh gene set contains genes up-regulated in aggressive uveal melanoma samples (GSEA [21], *q* < 10^-35^). These results may reflect the fact that we are more likely to identify cryptic 3’SSs in genes that are highly expressed which may bias such a gene set enrichment analysis. Nonetheless, several gene sets with potential importance for cancer development are enriched such as genes positively correlated with *BRCA1*, *ATM*, and *CHEK2* expression across normal tissues (GSEA, *q* < 10^-28^).

### Cryptic 3’SSs are located ~13–17 bp downstream of the branch point

**Fig 2:**
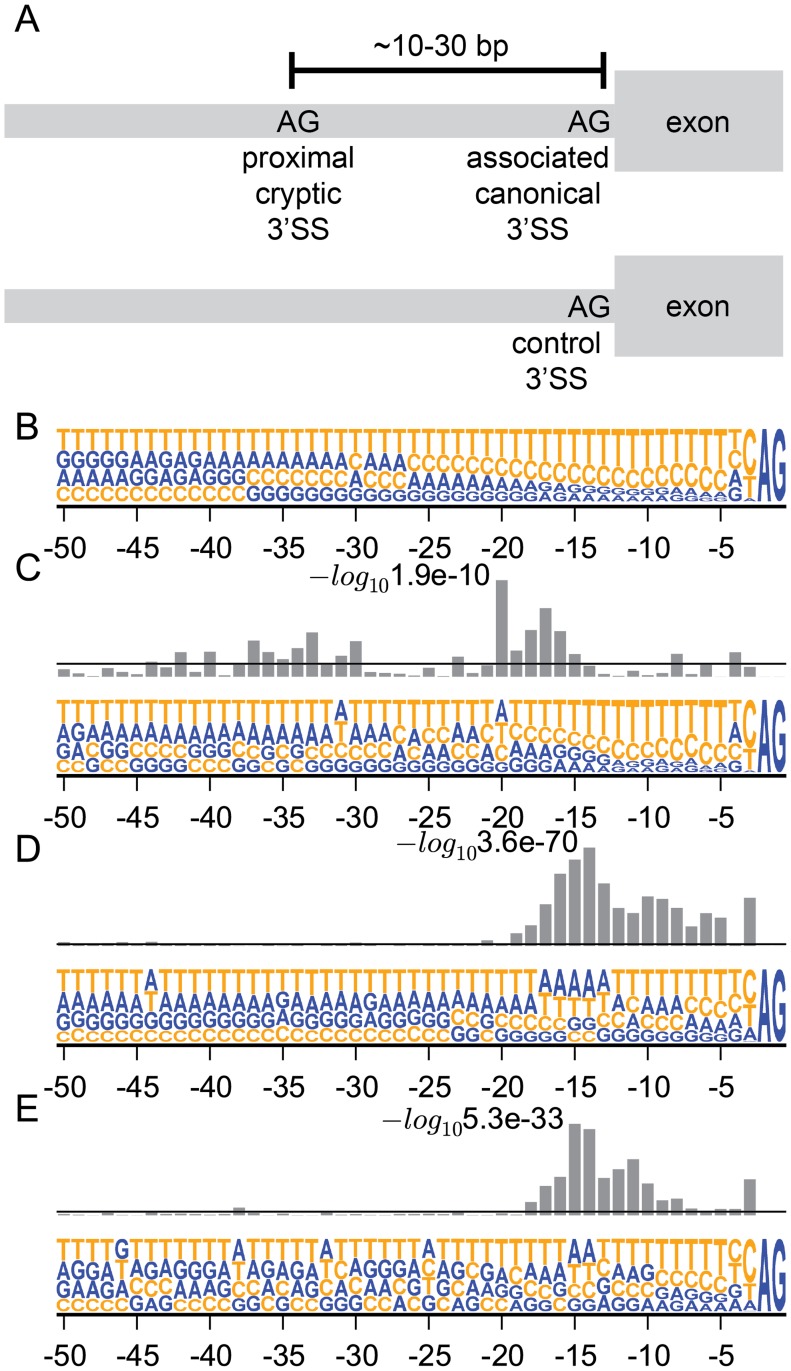
3’ intron nucleotide composition for control, associated canonical, and cryptic 3’SSs. (A) We identified 23,066 control 3’SSs whose junctions had a mean coverage greater than 100 reads over all CLL, BRCA, and UM samples to compare to the cryptic and associated canonical 3’SSs. Nucleotide frequency for the last 50 bp of the intron for (B) 23,066 control 3’SSs; (C) 613 associated canonical 3’SSs; (D) 619 proximal cryptic 3’SSs; and (E) 417 distal cryptic 3’SSs. Bar plots above each nucleotide composition plot are log10 *p-*values from Fisher exact tests for enrichment of adenines at each position relative to control 3’SSs. Horizontal line marks significance level of *p* = 0.05. (-log_10_ 0.05 ≈ 1.3). The *p*-value box plots have different scales in (C), (D), and (E); the smallest *p*-values for each panel are labeled.

**Fig 3:**
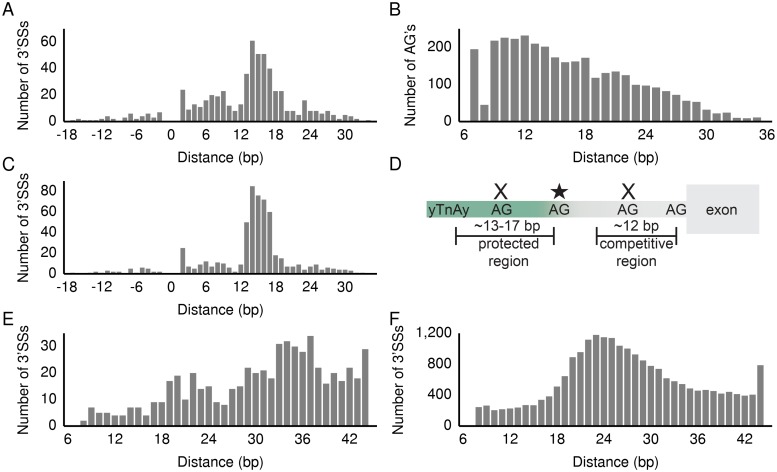
Location of predicted branch point relative to cryptic and canonical 3’SSs and model of cryptic 3’SS selection. (A) Distance from highest scoring BP predicted for associated canonical 3’SSs to the corresponding proximal cryptic 3’SSs. A negative distance indicates that the cryptic 3’SS is upstream of the BP predicted for the canonical 3’SS. The small spike at 2 bp indicates that in a few cases the adenine in the cryptic 3’SS is predicted to be the BP adenine for the canonical 3’SS. (B) Distance from highest scoring BP predicted for control 3’SSs to downstream intronic AG dinucleotides that are not annotated as 3’SSs. (C) Distance from either highest or second highest scoring BP predicted for canonical 3’SSs to their associated cryptic 3’SSs (see Methods). (D) Model for proximal cryptic 3’SS selection in *SF3B1* mutants. yTnAy is the human BP motif. AG dinucleotides located at the edge of the sterically protected region can be used as 3’SSs in *SF3B1* mutants (star). AG dinucleotides located in the protected or competitive regions (X’s) are respectively sterically hindered from being selected as 3’SSs or out-competed by the canonical 3’SS. Distance from predicted BP to 3’SS for (E) associated canonical 3’SSs and (F) control 3’SSs (see Methods) is significantly different (*p* < 10^-23^, Mann Whitney U).

We characterized the sequence features of the 619 proximal cryptic 3’SSs and their associated canonical 3’SSs to gain further insights into the mechanism of cryptic 3’SS selection (Fig. 2A). We chose 23,066 control 3’SSs (see Methods) and plotted the nucleotide frequency [22] for the last 50 bp of the introns for all control, associated canonical, and cryptic 3’SSs as well as the enrichment of adenines relative to the control introns. The control introns have a typical nucleotide composition with a 4–24 bp PPT preceding the 3’SS (Fig. 2B) [13]. The associated canonical 3’SS introns are enriched for adenines ~15–20 bp upstream of the 3’SS since the proximal cryptic 3’SSs are located in this region (Fig. 2C). However, the introns for proximal (Fig. 2D) and distal (Fig. 2E) cryptic 3’SSs have a strong enrichment of adenines concentrated ~15 bp upstream of the splice sites. These results suggest that the increased usage of the 619 proximal and 417 distal cryptic 3’SSs in the *SF3B1* mutants may result from the same mechanism. The human BP motif is highly degenerate except for a largely invariant adenine [13] leading us to suspect that the adenine signal upstream of the cryptic 3’SSs is caused by the associated canonical 3’SSs’ BP adenines. We used SVM_BP [23] to predict BPs for the associated canonical 3’SSs and calculated the distance from the highest scoring predicted BPs to the cryptic splice sites. We found that AG dinucleotides that serve as cryptic 3’SSs are enriched ~13–17 bp downstream from the predicted BP (Fig. 3A) relative to random AG dinucleotides present in control 3’SS introns (Fig. 3B, *p* < 10^-7^, Mann Whitney U). For cryptic 3’SSs not located 13–17 bp downstream from the highest scoring BP in Fig. 3A, we calculated the distance from the second highest scoring BP to the cryptic 3’SSs and found that overall, the majority of the cryptic 3’SSs were located 13–17 bp from either the highest or second highest scoring BP (Fig. 3C).

### Proposed mechanism of cryptic 3’SS selection

3’SSs are typically not located within ~12–18 bp downstream of the BP because the proteins bound to the BP sterically hinder AG dinucleotides in this region and prevent them from being used as 3’SSs [16]. Our results suggest that AG dinucleotides serving as cryptic 3’SSs in *SF3B1* mutants are located at the end of this sterically protected region downstream of the BP (Fig. 3D). Additionally, during the splicing reaction, the spliceosome searches ~12 bp downstream from the first 3’SS after the BP for any other 3’SSs and chooses the strongest 3’SS based on sequence features [16]. The lack of cryptic 3’SSs in the last 10 bp of the intron (Fig. 1A) indicates that cryptic 3’SSs used in *SF3B1* mutants are located far enough upstream of the associated canonical 3’SSs to avoid competition for splicing. We observed that the distance between associated canonical 3’SSs and their predicted BPs is significantly greater than the distance between control 3’SSs and their BPs such that the cryptic 3’SSs at the edge of the protected region do not compete with the canonical 3’SS for splicing (*p* < 10^-23^, Mann Whitney U, Fig. 3E,F). We also predicted BP’s for the 619 proximal and 417 distal cryptic 3’SSs (as opposed to above where we predicted BP’s for the canonical 3’SSs associated with the 619 proximal 3’SSs) and found that the majority of these cryptic 3’SSs were 13–17 bp downstream of their predicted BP’s (S5 Fig.) providing further evidence that most cryptic 3’SSs (both proximal and distal) associated with *SF3B1* mutations are located at the edge of the sterically protected region.

Our results suggest that the mechanism of cryptic 3’SS selection in *SF3B1* mutants is not altered BP recognition because a more varied distribution of distances from the cryptic 3’SS to the canonical 3’SS BP would be expected if BP recognition was altered. Studying the role of cryptic 3’SS in inherited Mendelian disease genes, Královicová *et al*. 2005 used splicing reporters with cryptic 3’SSs located in the PPT and found that moving the cryptic 3’SS into the ~12–18 bp sterically protected region reduced or eliminated cryptic 3’SS selection. On the other hand, moving an AG dinucleotide out of the sterically protected region allowed for its selection as a cryptic 3’SS [18]. These published experimental results and the rigid distance between the BP and the cryptic 3’SSs observed in our study are consistent with a model of altered 3’SS selection in *SF3B1* mutants due to a change in the size of the sterically hindered region downstream of the BP.

To test whether the sequences requirements defined here are sufficient for cryptic 3’SS usage, we identified 11,302 introns whose canonical 3’SSs passed our coverage cutoff of 20 reads summed over all samples and had potential cryptic 3’SSs (intronic AG dinucleotides that were 10–30 bp upstream of an annotated 3’SS and 13–17 bp downstream of the highest-scoring predicted BP). For 900 of these introns, the potential cryptic 3’SSs also passed the coverage cutoff, of which 310 were used significantly more often in the *SF3B1* mutants. This analysis demonstrates that not every potential cryptic 3’SS is differentially used in the mutants, so the sequence requirements described here appear to be necessary for cryptic 3’SS usage but not sufficient.

### Cryptic 3’SSs are used infrequently relative to canonical 3’SSs

**Fig 4:**
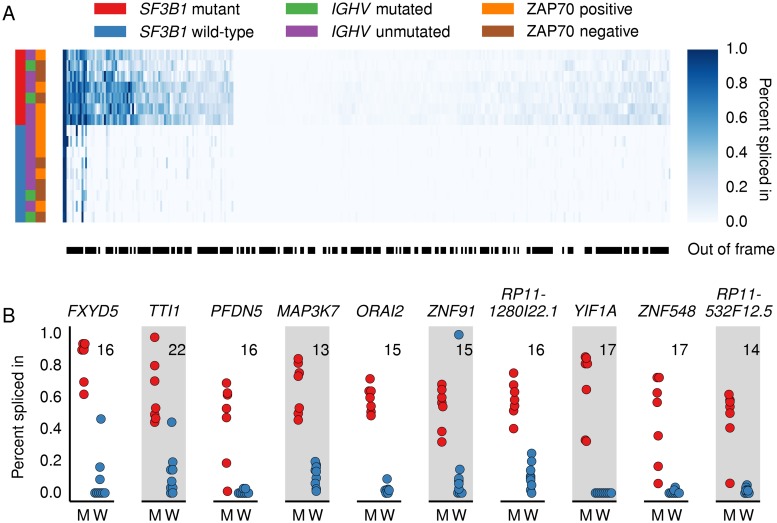
Percent spliced in for cryptic 3’ splice sites in CLL analysis. (A) Heatmap shows the percent spliced in (PSI) values for cryptic 3’SS relative to the canonical 3’SS in CLL *SF3B1* mutated or wild-type samples for 325 proximal cryptic 3’SSs used significantly more often in the CLL mutants (DEXSeq, BH-adjusted *p* < 0.1). *SF3B1* mutation presence and the status of prognostic factors *IGHV* and ZAP70 are shown in left colorbars. Black and white colorbar indicates whether novel 3’SSs are out-of-frame (black) relative to canonical 3’SSs. In-frame and out-of-frame cryptic 3’SSs are used at similar rates relative to their associated canonical 3’SSs. (B) Beeswarm plots indicating the PSI values for the cryptic 3’SS relative to the associated canonical 3’SS in ten genes with high levels of cryptic 3’SS inclusion in CLL *SF3B1* mutants (M) compared to wild-type (W) samples. No reads were observed spanning the cryptic *YIF1A* junction in any wild-type CLL samples. The number in the upper corner of each plot is the distance in base pairs from the highest or second-highest scoring BP predicted for the associated canonical 3’SS to the cryptic 3’SS.

Although the cryptic splice sites described here are used significantly more often in the *SF3B1* mutants, the biological effects are likely dependent on the proportion of transcripts that use the cryptic 3’SSs relative to the canonical 3’SSs. We therefore calculated the percent spliced in (PSI) for the proximal cryptic 3’SSs relative to their associated canonical 3’SSs in the CLL samples since they have a higher sequencing depth than the other tumor samples (S1 Fig.) that allows for more accurate quantification of splicing and because the distribution of well-characterized low- and high-risk CLL prognostic factors was similar between the *SF3B1* mutated and wild-type samples (Fig. 4A). To calculate PSI for the 325 proximal cryptic 3’SSs used significantly more often in the *SF3B1* mutants from the CLL-only analysis (S6–S7 Files), we divided the number of reads that span the cryptic 3’SS by the number of reads that span both the cryptic 3’SS and its associated canonical 3’SS. We observed that some cryptic 3’SSs are used exclusively in *SF3B1* mutants while others are also used in *SF3B1* wild-type samples but at a lower frequency relative to the mutants (Fig. 4A). 67% of the cryptic 3’SSs were included in <10% of transcripts compared to their associated canonical 3’SS. These results suggest that the cryptic splice sites are either included rarely even in the *SF3B1* mutants or that transcripts with cryptic splice sites are subject to a higher rate of nonsense-mediated decay (NMD). To investigate the potential role of NMD, we identified differentially expressed genes between the *SF3B1* mutant and wild-type samples in a joint analysis of all three cancers and performed a gene set enrichment analysis. We found that genes in the “Reactome NMD enhanced by the exon junction complex” set were enriched (GSEA [21], *q* < 10^-28^) among the 272 differentially expressed genes (DESeq2, BH-adjusted *p* < 0.1, S8–S9 Files) suggesting that NMD may be different between the *SF3B1* mutants and wild-types. 33 of the 582 genes that contained the 619 proximal cryptic 3’SSs were differentially expressed with the expression of 29/33 of these genes lower in the *SF3B1* mutants. Genes containing a proximal cryptic 3’SSs were more likely to be differentially expressed (Fisher exact, *p <* 10^-8^) and more likely to have lower expression in *SF3B1* mutants (Fisher exact, *p* = 0.0009). These results suggest that cryptic 3’SS selection may affect gene expression for a subset of genes. However, the observation that in-frame cryptic 3’SSs likely not subject to NMD and out-of-frame cryptic 3’SSs potentially subject to NMD are included at similar rates relative to their associated canonical 3’SSs (Fig. 4A) suggests that most genes’ expression are not affected by cryptic 3’SS selection and most cryptic 3’SSs are observed at a low frequency because they are spliced in infrequently compared to their associated canonical 3’SSs.

To identify cryptic 3’SSs with relatively high PSI values in the *SF3B1* mutant versus wild-type samples, we searched for cryptic 3’SSs that were 1) used more than 50% of the time in the CLL *SF3B1* mutants; 2) used less than 20% of the time in wild-type samples; and 3) had an average coverage of at least 30 junction-spanning reads in the mutant samples. Despite the generally low PSI values for the 325 cryptic 3’SSs from the CLL-only analysis, we identified four genes previously implicated in cancer (*TTI1* [24–26], *MAP3K7* [27–29], *FXYD5* [30], *PFDN5* [31]) and six others (*YIF1A*, *ORAI2*, *ZNF91*, *ZNF548*, *RP11–1280I22*.*1*, *RP11–532F12*.*5*) with out-of-frame cryptic 3’SSs that were consistently preferred to the associated canonical 3’SS in the CLL *SF3B1* mutant samples (Fig. 4B). Ferreira *et al*. identified the junctions in *ORAI2*, *ZNF91*, and *TTI1* in CLL *SF3B1* mutants as well [11]. Nine of the ten junctions were significant in our BRCA-only analysis and showed high differences in relative inclusion (S6 Fig., S10–S11 Files). These genes are not differentially expressed between the CLL *SF3B1* mutant and wild-type samples (S12 File) but the frequent inclusion of out-of-frame cryptic 3’SSs may affect their biological function.

## Discussion

Here we have shown that a consequence of *SF3B1* mutations in different cancer types is genome-wide selection of hundreds of cryptic 3’SSs. We have shown the cryptic 3’SSs have specific sequence requirements; AG dinucleotides used as cryptic 3’SSs in *SF3B1* mutants are located at the end of the sterically protected region ~13–17 bp downstream of the BP but are >10 bp upstream of nearby canonical 3’SSs allowing them to avoid competition for splicing. These sequence requirements limit the introns susceptible to cryptic 3’SS selection to those where the BP is located farther from the 3’SS than the typical ~24 bp. While these requirements appear necessary for cryptic 3’SS usage, they are not sufficient, as we did not detect cryptic 3’SS usage in all introns with AG dinucleotides that satisfy these requirements. Characteristics such as RNA conformation, RNA binding protein sites, BP prediction inaccuracies, cryptic or downstream canonical 3’SS strength, gene/transcript expression, sequencing depth, or other factors may also play a role in determining whether cryptic 3’SSs are used and detected by RNA sequencing.

Examining differential splice junction usage allowed us to identify many more cryptic 3’SSs than previous studies while still identifying 61 of 79 cryptic 3’SSs recently reported for CLL *SF3B1* mutants using a method based on relative inclusion [5,6,8,10,11]. When examining the three cancer types in our study individually, the number of cryptic 3’SSs identified was highly dependent on the sequencing depth of the samples (S1–S2 Figs., S2 File). Additionally, examining cryptic 3’SSs expressed higher in the *SF3B1* mutants but not significantly (Fig. 1B) shows a modest enrichment of novel 3’SSs 10–30 bp upstream of canonical 3’SSs. These observations suggest that deeper sequencing will continue to reveal proximal cryptic 3’SSs in *SF3B1* mutants that are used very infrequently or are present in lowly expressed genes.

Selection of cryptic 3’SSs in the region downstream of the BP has been reported for some inherited diseases including those resulting from disrupted tumor suppressor genes such as *ATM*, *NF1*, and *TP53* [18]. Using a curated a list of aberrant splice sites associated with different diseases from the literature, Královicová *et al*. 2005 found that in cases where cryptic 3’SS selection was not caused by mutation of the 3’YAG consensus sequence, cryptic 3’SSs were often located ~19 bp upstream of associated canonical 3’SSs and ~11–15 bp downstream of the BP [18]. Most of the diseases considered in Královicová *et al*. 2005 are Mendelian diseases where a cryptic 3’SS disrupts or abolishes the function of a single disease gene. In these cases, a mutation in the PPT between the sterically protected and competitive regions has introduced a cryptic 3’SS (Fig. 3D). For cancers with *SF3B1* mutations, we suspect that the size of the sterically protected region is slightly altered allowing for existing AG dinucleotides to be used as cryptic 3’SSs in hundreds of genes. It is also possible *SF3B1* mutations could cause destabilization of the U2 snRNP complex or alter interactions with U2AF2, affecting the ability to recognize the canonical 3’SS and leading to cryptic 3’SS selection. However, the rigid distance (~13–17 bp) from the predicted BPs to the cryptic 3’SSs for most of the cryptic 3’SSs is most consistent with a change in the size of the sterically protected region downstream of the branch point.

We found that cryptic 3’SS selection is limited to tumors with mutations in the five ~10 amino acid hotspots in the *SF3B1* HEAT 5–9 repeats and that these mutations are associated with cryptic 3’SS selection across different cancer types and even in cancers in which *SF3B1* is not recurrently mutated. 58% of these cryptic 3’SSs are out-of-frame relative to nearby canonical 3’SSs, but the biological impact of these cryptic 3’SSs is likely a function of how frequently they are used relative to the nearby canonical 3’SSs. We found that while the cryptic 3’SSs are used more often in the *SF3B1* mutated samples compared to wild-type samples, they are used relatively infrequently (<10%) compared to nearby canonical 3’SSs. While the differentially expressed genes between the *SF3B1* mutated and wild-type samples are enriched for genes in the NMD pathway, even in-frame cryptic 3’SSs are used at a low frequency indicating that the associated canonical 3’SS is mostly preferred to the cryptic 3’SS even in *SF3B1* mutants. Nonetheless, we identified ten genes, including four with known roles in cancer, which had a high frequency of cryptic splice site usage relative to the nearby canonical splice site. Further studies are required to determine whether low-frequency cryptic 3’SS selection in hundreds of genes, high-frequency cryptic 3’SS selection in a small group of genes, and/or other splicing alterations drive the oncogenic effect of *SF3B1* mutation.

## Methods

### Sample selection

**Ethics statement.** For the chronic lymphocytic leukemia (CLL) samples, the UCSD IRB approved the study and all subjects gave informed consent (Project #080918). Refer to the informed consent for The Cancer Genome Atlas and Harbour *et al*. for consent information for other cancer samples [7].

**CLL.** Seven *SF3B1*-mutated CLL cases and nine *SF3B1* wild-type CLL cases were identified from the CLL Consortium database. The mutations were originally characterized by PCR and verified in the RNA-sequencing data [9]. Sample dates were chosen on average 95 days prior to treatment and at least 287 days after prior treatment to select samples with high tumor cell count. Samples were chosen to have relatively similar numbers of *IGHV* mutated/unmutated and ZAP-70 positive/negative samples (Fig. 4).

**BRCA, LUAD, and LUSC.** *SF3B1* mutant samples were identified using the Broad GDAC TCGA analysis (http://gdac.broadinstitute.org/runs/analyses__2013_02_22/) in TCGA tumor types with no publication restrictions. Samples with *SF3B1* mutations outside of Gencode version 14 exons were excluded. We excluded any cancer types with less than four *SF3B1* mutants or for which paired-end RNA-sequencing data was not available leaving breast cancer (BRCA), lung adenocarcinoma (LUAD), and lung squamous cell carcinoma (LUSC). We chose 1.25 as many *SF3B1* wild-type controls as mutated samples for each cancer type randomly from samples without mutations in *SF3B1* or other splicing factors. RNA sequencing data was downloaded from CGHub [32].

**UM.** Uveal melanoma samples were downloaded from the Short Read Archive (SRA062359) [7]. As reported in Furney *et al*., four uveal melanoma samples had *SF3B1* mutations in codon 625 and four had wild-type copies of *SF3B1* [33].

### Library preparation and sequencing for CLL samples

RNA was extracted from peripheral blood mononucleocytes from seven *SF3B1-*mutated CLL cases and nine *SF3B1* wild-type cases per the manufacturer’s specifications using Qiagen RNeasy mini-spin columns, and RIN scores determined using an Agilent Bioanalyzer. RNA was polyA selected and processed using SMART cDNA synthesis (Clontech) to prepare sequencing libraries. Samples were sequenced on Illumina HiSeq2000 instruments generating an average of 239 million paired 75 bp reads per sample (S1 Fig.).

### Adapter trimming

Sequencing adapters and poly-A/T tails were trimmed for CLL samples only using cutadapt version 1.1 (-m 20—n 10—b AAGCAGTGGTATCAACGCAGAGTACTTTTTTTTTTT—b AAGCAGTGGTATCAACGCAGAGTACGCGGG—b AAGCAGTGGTATCAACGCAGAGT—b TTTTTTTTTTTTTTTTTTTTTTTTTTTTTTTTTTTTTTTTTTTTTTTTTTTTTTTTTTTTTTTTTTTTTTTTTTT—b AAAAAAAAAAAAAAAAAAAAAAAAAAAAAAAAAAAAAAAAAAAAAAAAAAAAAAAAAAAAAAAAAAAAAAAAAAA) [34]. Read pairs where or one of both reads were of length less than 20 were removed.

### Read alignment

RNA-seq reads were aligned to the human genome (hg19) using STAR 2.3.0e (—alignSJDBoverhangMin 1—seedSearchStartLmax 12—alignSplicedMateMapLminOverLmate 0.08—outFilterScoreMinOverLread 0.08—outFilterMatchNminOverLread 0.08—outFilterMultimapNmax 100—outFilterIntronMotifs RemoveNoncanonicalUnannotated—outSJfilterOverhangMin 6 6 6 6) and a splice junction database consisting of junctions from Gencode, UCSC knownGene, AceView, lincRNAs, and H-Inv [19,35–39]. Duplicate read pairs were removed prior to alignment by comparing the sequences of all read pairs and keeping only one read pair per set of read pairs with identical sequences.

### Splice junction read coverage

Splice junction read coverages were obtained from the SJ.out.tab output file from STAR.

### Novel splice junction identification

Novel splice junctions were defined as those junctions identified by STAR not present in Gencode version 14 that (i) were covered by at least 20 reads summed over all cancer samples in a given analysis, (ii) shared a 5’ splice site and/or 3’SS with a Gencode junction, and (iii) had one of the following motifs: GU/AG, CU/AC, GC/AG, CU/GC, AU/AC, GU/AU. Novel junctions were calculated separately for each analysis.

### Splice junction usage

Known and novel junctions that had a coverage of at least 20 reads over all samples, used a known intron motif, and contained a known Gencode 5’ splice site or 3’SS were aggregated by gene and tested for differential usage using DEXSeq’s testForDEUTRT function (v1.8.0, R v3.0.3) [20]. Splice junctions used in more than one Gencode gene were removed. When multiple cancer types were analyzed, we provided cancer type as a covariate to DEXSeq. Raw *p-*values were adjusted for multiple hypothesis testing using the Benjamini Hochberg procedure. To examine the impact of the coverage cutoff of 20 reads summed over all samples on our results, we increased the cutoff to 50, 75, and 100 reads summed over all samples and found that 42%, 32%, and 24% of the significant novel 3’SSs remained at each of these cutoffs. The enrichment for proximal cryptic 3’SS remained at all cutoffs, so we used the 20 read cutoff to maximize sensitivity.

### Identification of associated canonical 3’SSs for cryptic 3’SSs

Associated canonical 3’SSs were identified for novel/cryptic 3’SSs as follows. First, all Gencode splice sites that shared a 5’ splice site with the novel 3’SS were identified. Then, the closest Gencode 3’SS from these splice sites that was downstream of the cryptic 3’SS was chosen as the associated canonical 3’SS for that cryptic 3’SS. If there was no Gencode 3’SS downstream of the cryptic 3’SS, the closest Gencode 3’SS upstream of the cryptic 3’SS was chosen as the associated canonical 3’SS.

### Gene set enrichment for genes with cryptic 3’SS usage

We performed a gene set enrichment analysis using GSEA [21] for the genes that contained cryptic 3’SSs by combining the genes that contained the 619 proximal (S3 File) and the 417 distal cryptic 3’SSs (S4 File).

### Identification of control 3’SSs

We identified 23,066 control 3’SSs by choosing splice sites that are annotated in Gencode, whose average coverage over BRCA, CLL, and UM samples is greater than 100, and whose 5' splice site does not have any novel 3'SSs. We characterized intronic AG dinucleotides for these control junctions by analyzing the intronic sequence downstream of the predicted branch points minus the last 10 bp of the intron since alternative 3’SSs can be located in the last 10 bp of the intron.

### Hierarchical clustering

All heatmap rows and columns were clustered using scipy.cluster.hierarchy.linkage with either the “complete” or “single” distance metric.

### *SF3B1* mutant allele frequency

Mutant allele frequency was determined by calculating per-base coverages using unique properly paired reads with samtools mpileup for the *SF3B1* locus and counting the number of reads supporting either the reference or alternate alleles.

### Gene expression

Reads that were not contained within Gencode v14 exons in the STAR genomic alignment were discarded. The remaining reads were re-aligned to the Gencode v14 transcriptome using Bowtie2 (v2.1.0,-t-k 400-X 400—no-mixed—no-discordant) and transcript expression was estimated using eXpress (v1.3.0,—max-indel-size 20) [40,41]. Gene expression was estimated by summing together the effective counts or FPKM values for all transcripts contained in a gene.

### Relative average expression of genes with cryptic 3’SSs

For the green heatmap in Fig. 1D, the average expression (FPKM) of each gene containing a cryptic 3’SS was determined for each cancer type. The average expression values were then normalized for each gene by dividing by the largest average expression of the three cancers for that gene. Therefore each column in the green heatmap in Fig. 1D has one value of 1.0 while the other two values are between 0.0 and 1.0 and represent the expression of the gene in that cancer relative to the maximum.

### Definition of HEAT repeats

HEAT repeat locations were defined according to the definition of HEAT repeats in Wang *et al*. 1998 [15].

### COSMIC SF3B1 mutations

COSMIC v66 complete export was downloaded and the number of mutations at each location in the *SF3B1* heat domains 5–9 was plotted for locations with at least two observed mutations in COSMIC [42].

### Nucleotide frequency plots

Nucleotide frequency plots were constructed using WebLogo (unit_name = ’probability’) [22]. Adenine enrichment was calculated by counting the number of adenines and non-adenines at each intron position for a given splice site class and comparing to the number of adenines and non-adenines in control 3’SSs using a Fisher exact test.

### Branch point identification

SVM_BP was used to predict branch points [23]. The SVM_BP code was altered to allow for branch points eight bp from the 3’SS by setting mindist3ss = 8 in svm_getfeat.py (see https://github.com/cdeboever3/svm-bpfinder). SVM_BP was run with options “Hsap 50.” When multiple branch points were predicted for one 3’SS, we chose the branch point with the highest sequence score (bp_scr). In some instances, there was more than one cryptic 3’SS associated with a canonical 3’SS, so we randomly chose only one of these cryptic splice sites for further analysis. For Fig. 3C, we plotted the distance from highest scoring BP predicted for canonical 3’SSs to their associated cryptic 3’SSs as in Fig. 3A. However, the distances for cryptic 3’SSs located less than 13 bp or more than 17 bp from the BP in Fig. 3A were replaced with the distance from the second highest scoring BP. S5C–S5D Fig. were created similarly.

### Differential gene expression

Gene expression was estimated as described above. We summed the effective counts from eXpress for all transcripts from each gene to obtain effective read counts for each gene. We provided these read counts to DESeq2 (v1.2.10, R v3.0.3) and tested for differential gene expression using nbinomWaldTest using cancer type as a covariate for the analysis with different cancers [43]. We only tested genes where the sum of effective read counts over all samples was greater than 100. *p*-values were adjusted using the Benjamini-Hochberg procedure. Gene set enrichment analysis was performed using GSEA [21].

### Percent spliced in for cryptic 3’SSs relative to associated canonical 3’SSs

Percent spliced in (PSI) values for cryptic 3’SSs relative to canonical 3’SSs were calculated by dividing the number of reads that span the cryptic 3’SS (*c*) by the number of reads that span the cryptic 3’SS plus the number of reads that span the canonical 3’SS (*a*), $\frac{c}{c+a}$, for each sample. The ten 3’SSs with high PSI values in CLL were identified by identifying cryptic 3’SSs whose median PSI was greater than 50% in the CLL *SF3B1* mutants but less than 20% in the wild-type samples and whose average coverage was at least 30 junction-spanning reads in the CLL mutant samples. These junctions were also chosen to be out-of-frame although the cryptic 3’SS in *ORAI2* is located in the 5’ untranslated region.

### Code, data, and reproducibility

We have made the code and intermediate data files needed to replicate this study available on Github (https://github.com/cdeboever3/deboever-sf3b1-2015) and Figshare (http://dx.doi.org/10.6084/m9.figshare.1120663). Instructions are provided in the Github repository for reproducing our figures, tables, and statistical analyses. Sequencing data is available through dbGaP (phs000767).

## Supporting Information

S1 Fig Number of uniquely mapped RNA-seq reads from STAR alignment. We sequenced the transcriptomes of peripheral blood mononucleocytes from seven *SF3B1-*mutated chronic lymphocytic leukemia (CLL) cases and nine *SF3B1* wild-type cases. We also obtained data from breast cancer (BRCA; 14 mutant, 18 wild-type), lung squamous cell carcinoma (LUSC; four mutant, five wild-type) and lung adenocarcinoma (LUAD; seven mutant, nine wild-type) samples from the TCGA and uveal melanoma (UM; four mutant, four wild-type) samples from Harbour *et al*. 2013.
(TIF)

S2 Fig Proximal cryptic 3’SSs in individual cancer analyses. log_2_ distance in base pairs from 280, 1,476, and 86 significantly differentially used novel 3’SSs (S2 File) to their associated canonical 3’SSs in (A) BRCA, (B) CLL, and (C) UM analyses respectively. Novel 3’SSs were associated with canonical 3’SSs only if they shared the same 5’ splice site. Zero represents the position of the canonical 3’SS. Negative and positive distances indicate that the cryptic 3’SS is respectively upstream or downstream from the canonical 3’SS. Inset shows base-by-base binning from zero to 50 base pairs upstream of canonical 3’SS. Red and blue histograms represent junctions with significantly higher usage in *SF3B1* mutants or *SF3B1* wild-type samples respectively. The number of cryptic 3’SS identified varied with the overall sequencing depth of the different data sets.
(TIF)

S3 Fig Breast cancer proximal cryptic 3’SS coverage. Heatmap shows for each BRCA sample the log_2_ library-normalized count *z*-score for 192 proximal cryptic 3’SSs used significantly more often in the *SF3B1* mutants and located 10–30 bp upstream of canonical 3’SSs (S2 File). *SF3B1* mutants are labeled with the observed missense or nonsense (*) mutation as well as the frequency of the mutant allele in the RNA-sequencing data. Attenuated cryptic 3’SS selection is visible for the K700E mutant with only 8.4% allele frequency. A633V and Y765C mutants do not show evidence for cryptic 3’SS selection. Black and white colorbar indicates whether novel 3’SSs are out-of-frame (black) relative to canonical 3’SSs.
(TIF)

S4 Fig Proximal cryptic 3’SSs used significantly more often in cancers with *SF3B1* hotspot mutations including TCGA lung cancer samples. Heatmap shows for each sample the log_2_ library-normalized count *z*-score for the 578 proximal cryptic 3’SSs used significantly more often in the *SF3B1* mutants in the CLL, BRCA, UM, LUAD, and LUSC joint analysis (S2 File). Grey bars indicate frequency of *SF3B1* mutant allele in RNA-seq data. Colorbars indicate *SF3B1* mutation status, cancer type, and whether the *SF3B1* mutation is located in the HEAT 5–9 repeats. Black and white colorbar indicates whether novel 3’SSs are out-of-frame (black) relative to canonical 3’SSs.
(TIF)

S5 Fig Cryptic 3’SSs have branch points located ~13–17 bp upstream. Distance from 3’SS to highest scoring predicted branch point (BP). We were able to predict BPs for (A) 584 of 619 proximal cryptic 3’SSs and (B) 405 of 417 distal cryptic 3’SSs (as opposed to predicting the BPs for the associated canonical 3’SSs as in Fig. 3). Distance from either highest or second highest scoring predicted BP to (C) proximal cryptic 3’SSs and (D) distal cryptic 3’SSs. Cryptic 3’SSs that are used more often in *SF3B1* mutants have BPs located ~13–17 bp upstream regardless of whether they are 10–30 bp upstream of canonical 3’SSs.
(TIF)

S6 Fig Percent spliced in (PSI) in BRCA analysis for junctions with high PSI in CLL analysis. Beeswarm plots showing the PSI values for the cryptic 3’SS relative to the associated canonical 3’SS in nine of ten genes with high levels of cryptic 3’SS inclusion in CLL *SF3B1* mutants (M) compared to wild-type (W) samples that were also expressed in the BRCA samples. The number in the upper corner of each plot is the distance in base pairs from the highest or second-highest scoring BP predicted for the associated canonical 3’SS to the cryptic 3’SS.
(TIF)

S1 File Metadata for samples used in this study. *SF3B1* mutated samples have columns for frequency of *SF3B1* mutation in RNA-seq data, mutation type, codon change and whether the mutation is in the HEAT 5–9 repeats. These columns are empty for *SF3B1* wild-type tumor samples.
(TSV)

S2 File Summary of differential junction usage results from DEXSeq. DEXSeq was used to test for differential splice junction usage in a joint analysis of the CLL, BRCA, and UM samples as well as individually for each cancer type. “Novel” indicates that the junction is not annotated in Gencode. Proximal indicates that a novel 3’SS is 10–30 bp upstream of a canonical Gencode 3’SS.
(TSV)

S3 File 619 cryptic 3’SSs located 10–30 bp upstream of canonical 3’SSs from joint BRCA, CLL, and UM analysis. Location of 5’ splice sites and 3’SSs are one-based coordinates that denote the start and end of the intron. The columns COSMIC, TSgene, and ncg denote whether the gene is present in COSMIC, TSGene, or the Network of Cancer Genes respectively.
(TSV)

S4 File 417 distal cryptic 3’SSs used more often in *SF3B1* mutants from joint BRCA, CLL, and UM analysis. Location of 5’ splice sites and 3’SSs are one-based coordinates that denote the start and end of the intron. The columns COSMIC, TSgene, and ncg denote whether the gene is present in COSMIC, TSGene, or the Network of Cancer Genes respectively.
(TSV)

S5 File GSEA results for 912 genes containing 619 proximal and 417 distal cryptic 3’ splice sites used more often in *SF3B1* mutants. (XLS)

S6 File 325 significant cryptic 3’SSs located 10–30 bp upstream of canonical 3’SSs and used more often in *SF3B1* mutants from CLL-only DEXSeq analysis. Location of 5’ splice sites and 3’SSs are one-based coordinates that denote the start and end of the intron. The columns COSMIC, TSgene, and ncg denote whether the gene is present in COSMIC, TSGene, or the Network of Cancer Genes respectively.
(TSV)

S7 File Percent spliced in for 325 cryptic 3’SSs located 10–30 bp upstream of canonical 3’SSs from CLL-only DEXSeq analysis. Note that there are only 324 values because one canonical 3’SS was filtered due to low coverage so a PSI value could not be calculated.
(TSV)

S8 File 272 genes that are differentially expressed between *SF3B1* mutant and wild-type samples from joint analysis of CLL, BRCA, and UM using DESeq2. (TSV)

S9 File GSEA results for 272 genes differentially expressed genes from joint CLL, BRCA, and UM DESeq2 analysis. (XLS)

S10 File 192 significant cryptic 3’SSs located 10–30 bp upstream of canonical 3’SSs and used more often in *SF3B1* mutants from BRCA-only DEXSeq analysis. Location of 5’ splice sites and 3’SSs are one-based coordinates that denote the start and end of the intron. The columns COSMIC, TSgene, and ncg denote whether the gene is present in COSMIC, TSGene, or the Network of Cancer Genes respectively.
(TSV)

S11 File Percent spliced in for 192 cryptic 3’SSs located 10–30 bp upstream of canonical 3’SSs from BRCA-only DEXSeq analysis. Note that there are only 191 values because one canonical 3’SS was filtered due to low coverage so a PSI value could not be calculated.
(TSV)

S12 File 33 genes that are differentially expressed between *SF3B1* mutant and wild-type CLL samples using DESeq2. (TSV)
