# Supplementary figures and images for: Transcriptome Sequencing Reveals Potential Mechanism of Cryptic 3’ Splice Site Selection in SF3B1-mutated Cancers
Source: PLoS Comput Biol. 2015 Mar 13;11(3):e1004105. doi: 10.1371/journal.pcbi.1004105 (PMC4358997; doi:10.1371/journal.pcbi.1004105)

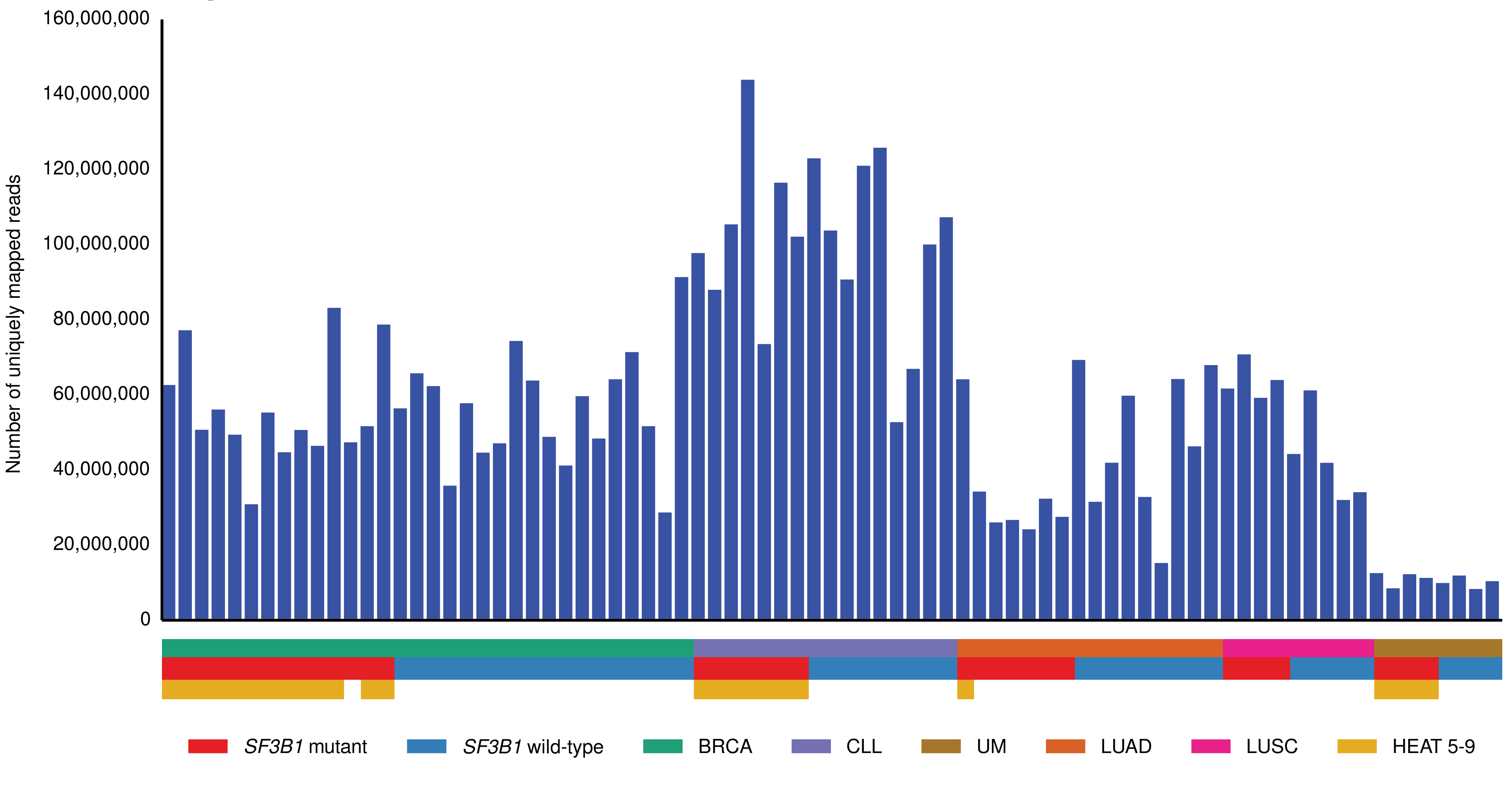

Supplement: S1 Fig — We sequenced the transcriptomes of peripheral blood mononucleocytes from seven SF3B1-mutated chronic lymphocytic leukemia (CLL) cases and nine SF3B1 wild-type cases. We also obtained data from breast cancer (BRCA; 14 mutant, 18 wild-type), lung squamous cell carcinoma (LUSC; four mutant, five wild-type) and lung adenocarcinoma (LUAD; seven mutant, nine wild-type) samples from the TCGA and uveal melanoma (UM; four mutant, four wild-type) samples from Harbour et al. 2013. (TIF) [file pcbi.1004105.s001.tif]

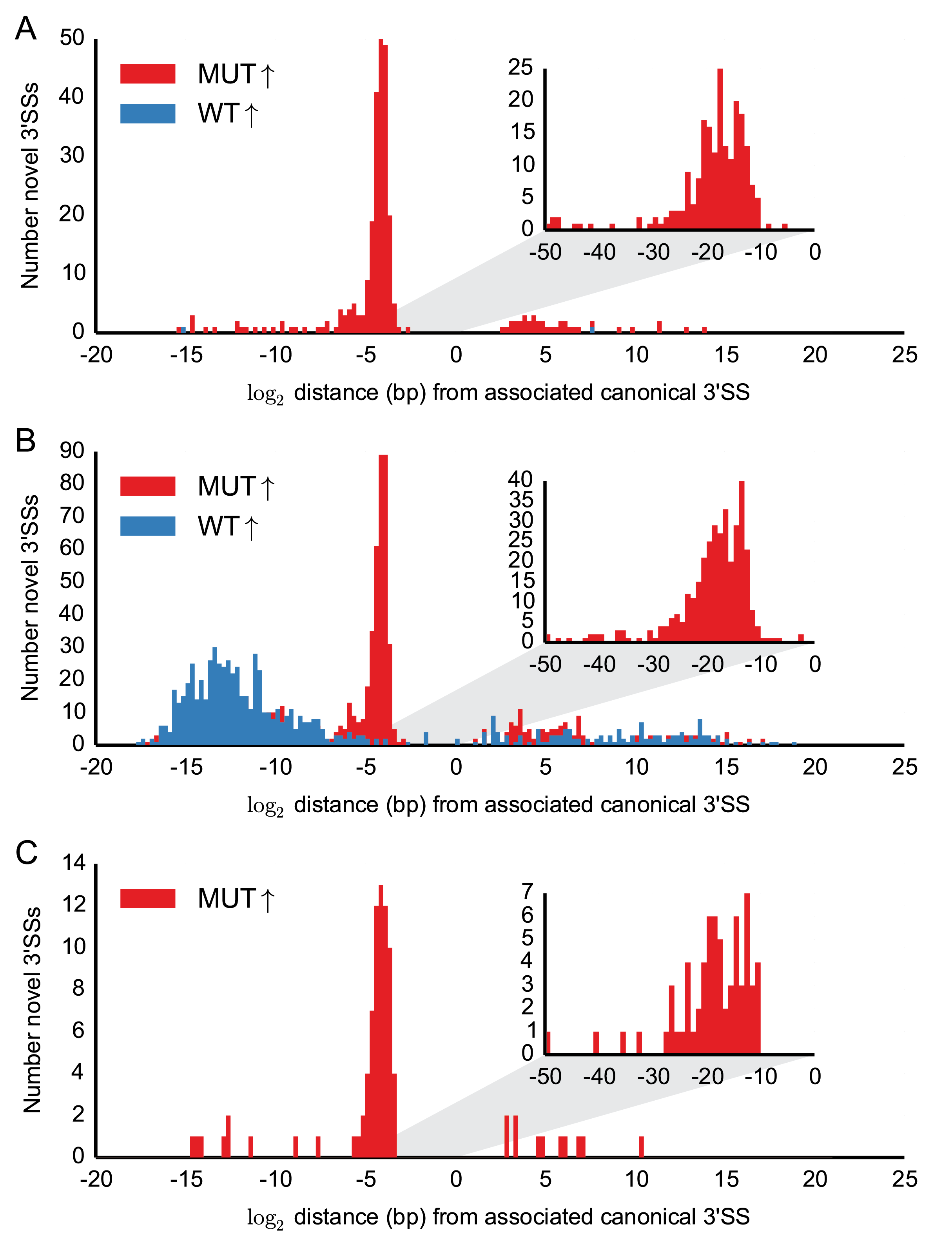

Supplement: S2 Fig — log2 distance in base pairs from 280, 1,476, and 86 significantly differentially used novel 3’SSs (S2 File) to their associated canonical 3’SSs in (A) BRCA, (B) CLL, and (C) UM analyses respectively. Novel 3’SSs were associated with canonical 3’SSs only if they shared the same 5’ splice site. Zero represents the position of the canonical 3’SS. Negative and positive distances indicate that the cryptic 3’SS is respectively upstream or downstream from the canonical 3’SS. Inset shows base-by-base binning from zero to 50 base pairs upstream of canonical 3’SS. Red and blue histograms represent junctions with significantly higher usage in SF3B1 mutants or SF3B1 wild-type samples respectively. The number of cryptic 3’SS identified varied with the overall sequencing depth of the different data sets. (TIF) [file pcbi.1004105.s002.tif]

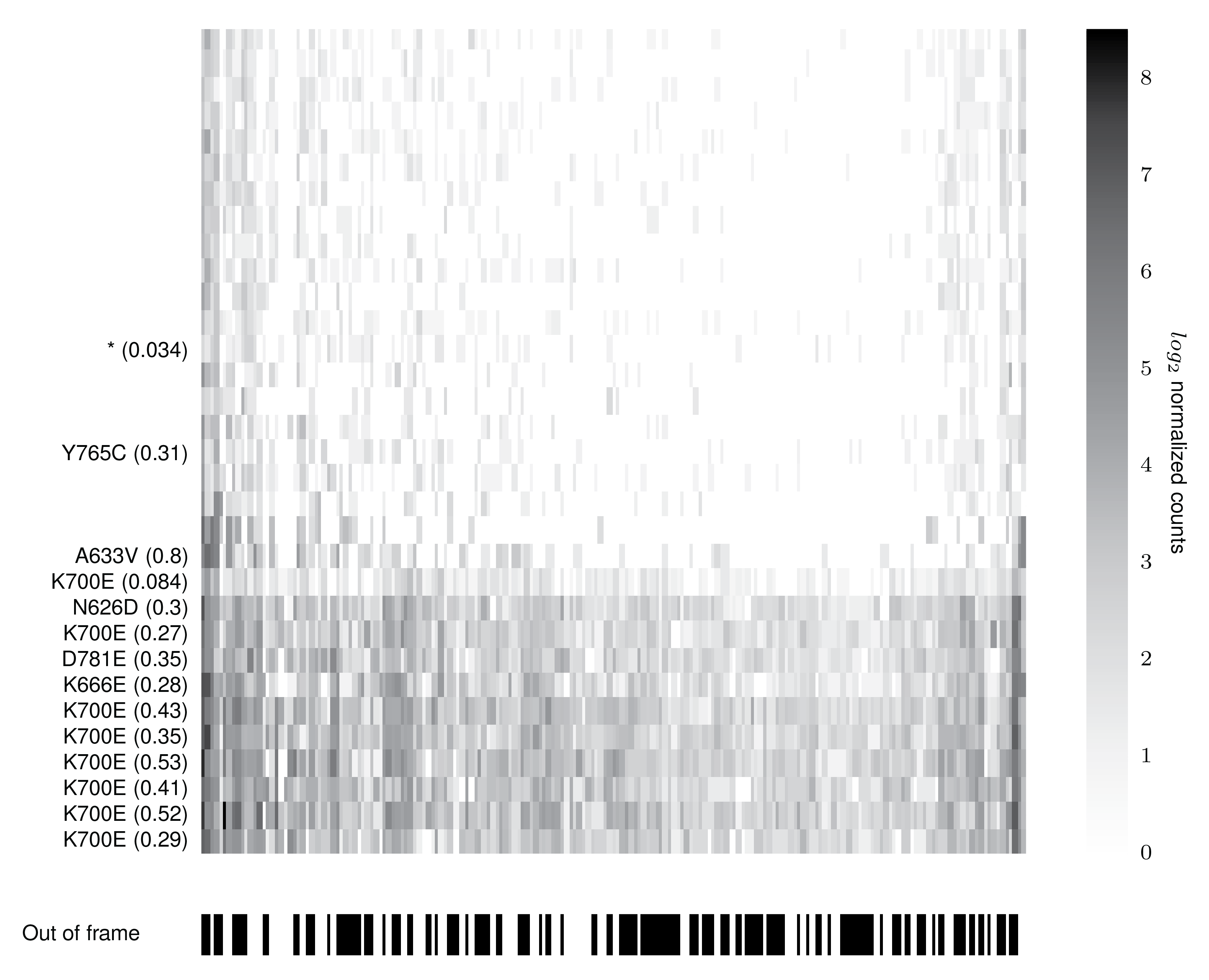

Supplement: S3 Fig — Heatmap shows for each BRCA sample the log2 library-normalized count z-score for 192 proximal cryptic 3’SSs used significantly more often in the SF3B1 mutants and located 10–30 bp upstream of canonical 3’SSs (S2 File). SF3B1 mutants are labeled with the observed missense or nonsense (*) mutation as well as the frequency of the mutant allele in the RNA-sequencing data. Attenuated cryptic 3’SS selection is visible for the K700E mutant with only 8.4% allele frequency. A633V and Y765C mutants do not show evidence for cryptic 3’SS selection. Black and white colorbar indicates whether novel 3’SSs are out-of-frame (black) relative to canonical 3’SSs. (TIF) [file pcbi.1004105.s003.tif]

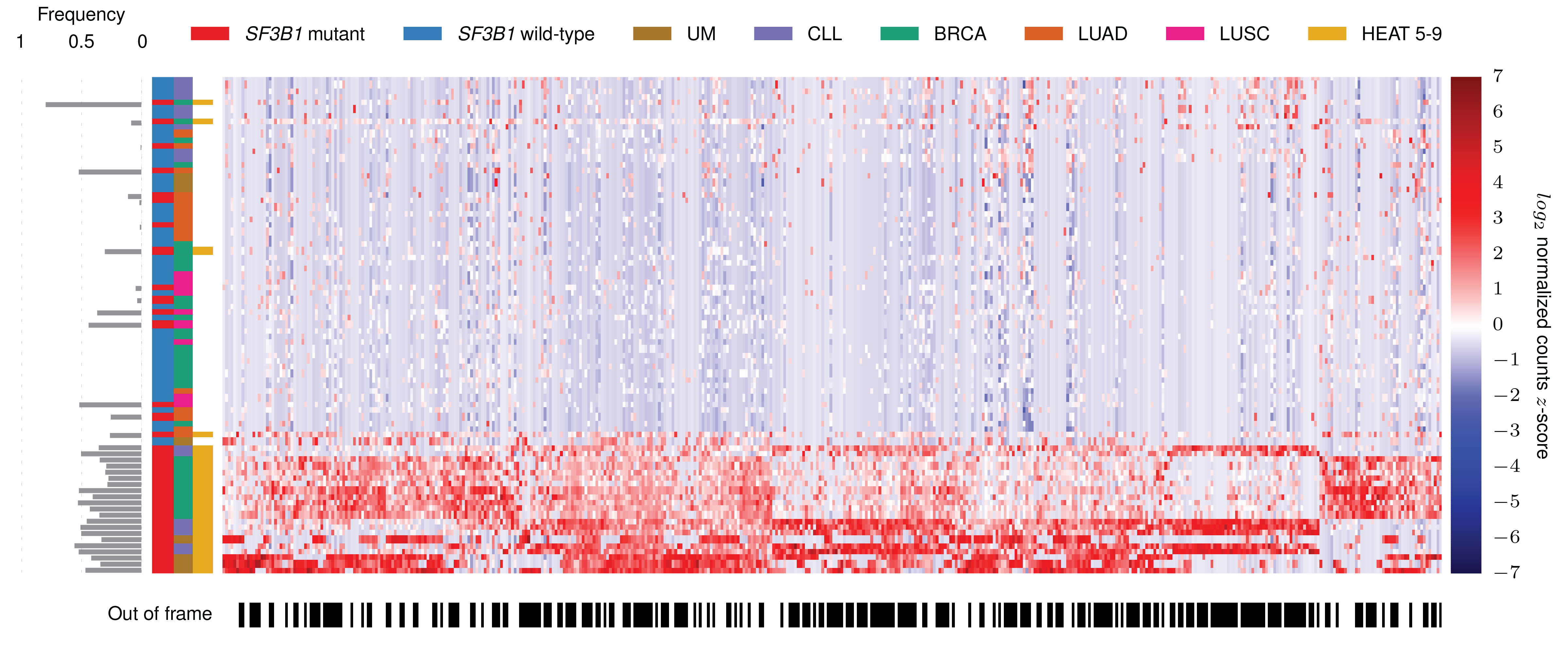

Supplement: S4 Fig — Heatmap shows for each sample the log2 library-normalized count z-score for the 578 proximal cryptic 3’SSs used significantly more often in the SF3B1 mutants in the CLL, BRCA, UM, LUAD, and LUSC joint analysis (S2 File). Grey bars indicate frequency of SF3B1 mutant allele in RNA-seq data. Colorbars indicate SF3B1 mutation status, cancer type, and whether the SF3B1 mutation is located in the HEAT 5–9 repeats. Black and white colorbar indicates whether novel 3’SSs are out-of-frame (black) relative to canonical 3’SSs. (TIF) [file pcbi.1004105.s004.tif]

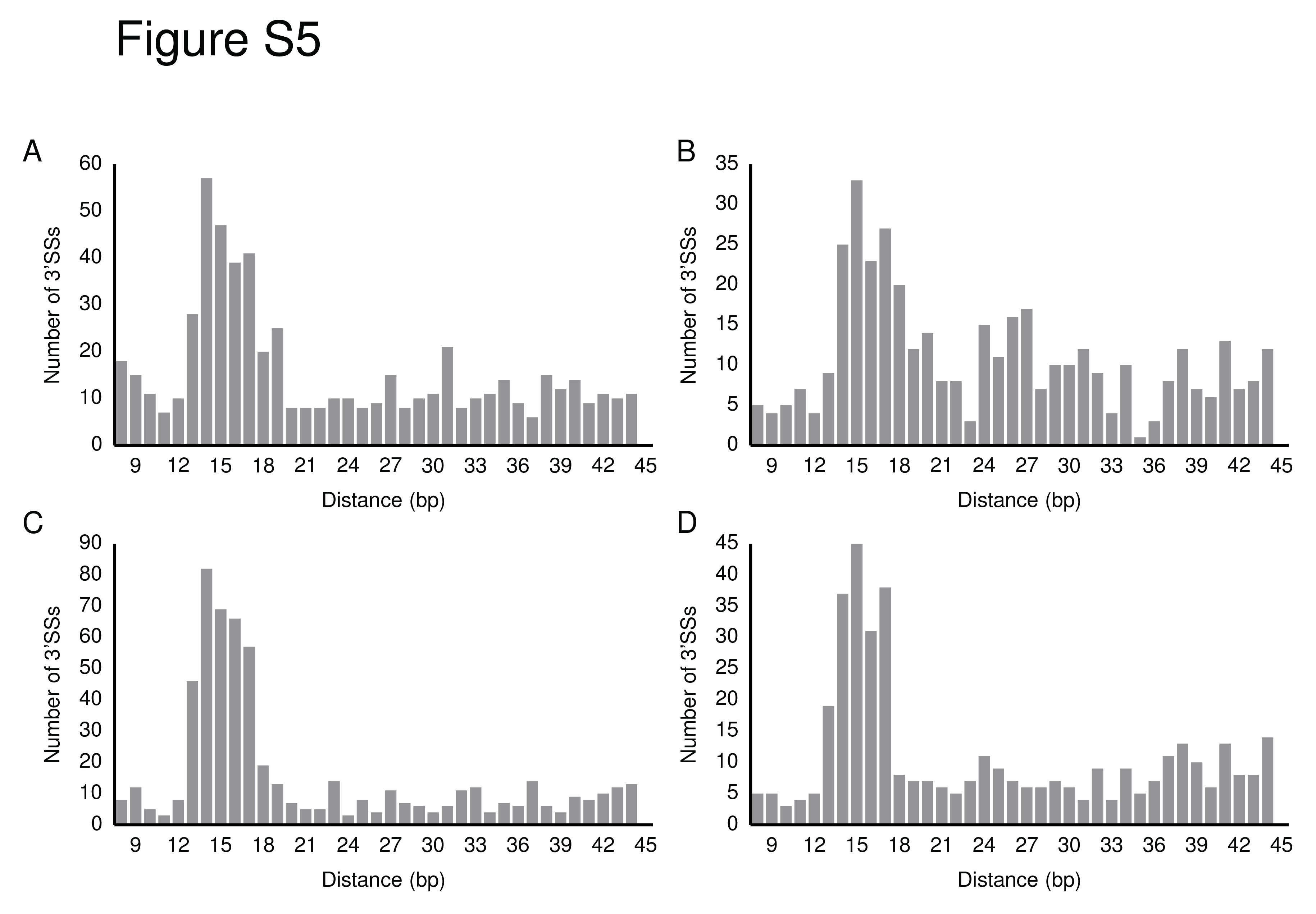

Supplement: S5 Fig — Distance from 3’SS to highest scoring predicted branch point (BP). We were able to predict BPs for (A) 584 of 619 proximal cryptic 3’SSs and (B) 405 of 417 distal cryptic 3’SSs (as opposed to predicting the BPs for the associated canonical 3’SSs as in Fig. 3). Distance from either highest or second highest scoring predicted BP to (C) proximal cryptic 3’SSs and (D) distal cryptic 3’SSs. Cryptic 3’SSs that are used more often in SF3B1 mutants have BPs located ~13–17 bp upstream regardless of whether they are 10–30 bp upstream of canonical 3’SSs. (TIF) [file pcbi.1004105.s005.tif]

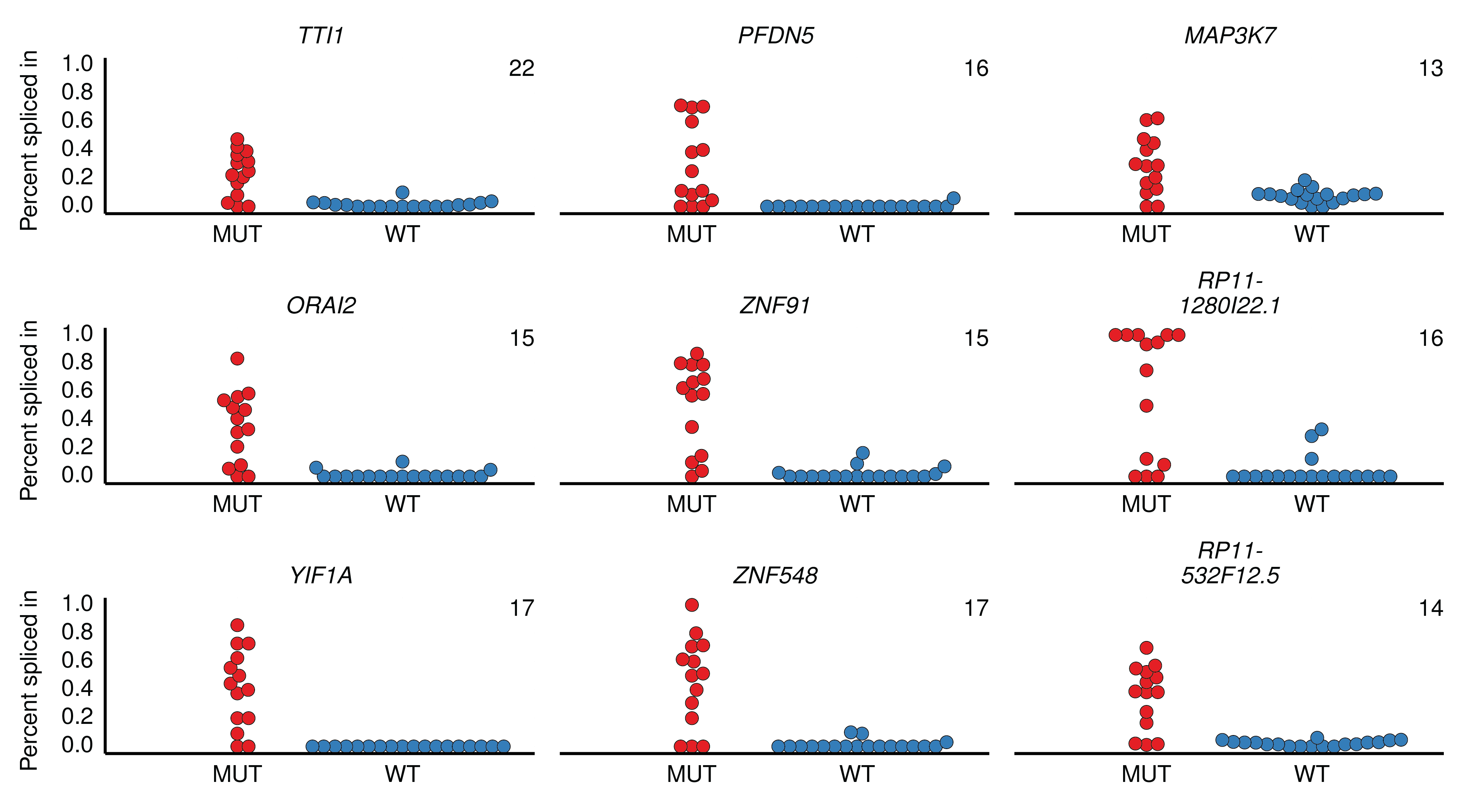

Supplement: S6 Fig — Beeswarm plots showing the PSI values for the cryptic 3’SS relative to the associated canonical 3’SS in nine of ten genes with high levels of cryptic 3’SS inclusion in CLL SF3B1 mutants (M) compared to wild-type (W) samples that were also expressed in the BRCA samples. The number in the upper corner of each plot is the distance in base pairs from the highest or second-highest scoring BP predicted for the associated canonical 3’SS to the cryptic 3’SS. (TIF) [file pcbi.1004105.s006.tif]
